# Exfoliative Toxins of *Staphylococcus aureus*

**DOI:** 10.3390/toxins2051148

**Published:** 2010-05-25

**Authors:** Michal Bukowski, Benedykt Wladyka, Grzegorz Dubin

**Affiliations:** 1 Department of Analytical Biochemistry, Faculty of Biochemistry, Biophysics and Biotechnology, Jagiellonian University, Krakow, Poland; Email: mbukow@gmail.com (M.B.); wladykab@interia.pl (B.W.); 2 Department of Microbiology, Faculty of Biochemistry, Biophysics and Biotechnology, Jagiellonian University, Krakow, Poland

**Keywords:** exfoliative toxin, epidermolytic toxin, *Staphylococcus aureus*, staphylococcal scalded skin syndrome, bullous impetigo

## Abstract

*Staphylococcus aureus* is an important pathogen of humans and livestock. It causes a diverse array of diseases, ranging from relatively harmless localized skin infections to life-threatening systemic conditions. Among multiple virulence factors, staphylococci secrete several exotoxins directly associated with particular disease symptoms. These include toxic shock syndrome toxin 1 (TSST-1), enterotoxins, and exfoliative toxins (ETs). The latter are particularly interesting as the sole agents responsible for staphylococcal scalded skin syndrome (SSSS), a disease predominantly affecting infants and characterized by the loss of superficial skin layers, dehydration, and secondary infections. The molecular basis of the clinical symptoms of SSSS is well understood. ETs are serine proteases with high substrate specificity, which selectively recognize and hydrolyze desmosomal proteins in the skin. The fascinating road leading to the discovery of ETs as the agents responsible for SSSS and the characterization of the molecular mechanism of their action, including recent advances in the field, are reviewed in this article.

## 1. Introduction

*Staphylococcus aureus* is a dangerous human pathogen responsible for a wide variety of diseases. Unlike the virulence of many bacteria, which is primarily dependent on the production of a single or limited number of virulence factors to which the observed clinical symptoms can be directly attributed, staphylococci secrete a wide spectrum of diverse extracellular proteins, which render the bacterium virulent. Although these factors, as a group, are essential for staphylococcal virulence, they largely lack the characteristics of typical toxins. They do not act alone, causing specific symptoms, when purified and administered in the absence of the bacterium, and the bacterial virulence is not markedly reduced when only a single factor is knocked out. Nonetheless, some symptoms associated with *S. aureus* infection are caused by typical toxins, such as toxic shock syndrome toxin 1 (TSST-1), enterotoxins, and exfoliative toxins (ETs) [1,2]. Exfoliative toxins (also known as “epidermolytic” toxins) are particularly interesting virulence factors of *S. aureus*. These extremely specific serine proteases recognize and cleave desmosomal cadherins only in the superficial layers of the skin, which is directly responsible for the clinical manifestation of staphylococcal scalded skin syndrome (SSSS). In this review, the reader is given a brief historic perspective on the fascinating road leading to the discovery of ETs, followed by a description of the present state of the art and the most recent developments in the characterization of the molecular mechanisms underlying ET functions. Finally, directions for further research are proposed.

## 2. Staphylococcal Scalded Skin Syndrome (SSSS)

Staphylococcal scalded skin syndrome, also known as Ritter’s disease, is primarily characterized by skin exfoliation [3,4]. Early SSSS manifests with fever, malaise, lethargy, and poor feeding. These symptoms are followed by an erythematous rash and the formation of large, fragile, fluid-filled blisters. The blisters burst with mechanical action, leaving the affected parts of the body without a protective layer of epidermis [5,6]. Only the skin, but not the mucosa, is involved [7]. SSSS affects large parts of the body and the lesions are often sterile. A localized form of SSSS, restricted to the sites of infection, is recognized as “bullous impetigo”. Both conditions share the same etiology and differ only in the extent of skin damage.

A diagnosis must distinguish SSSS from other skin diseases, such as toxic epidermal necrolysis, epidermolysis bullosa, bullous erythema multiforme, or listeriosis, and thermal or chemical burns, all of which can manifest with similar symptoms [5]. The simplest and most suitable methods of routine diagnosis are PCR for toxin-encoding genes or random amplified polymorphic DNA analysis [8,9,10]. Successful treatment is generally limited to the administration of intravenous antibiotics [3,11], and resistance is not yet a major problem. The prevalence of ETA does not differ significantly among methicillin-resistant (MRSA) and methicillin-susceptible (MSSA) strains. Recent reports demonstrated that 3-4% of MSSA strains carry the *eta* or *etb* gene [12,13], whereas around 10% of MRSA are *eta* positive [13]. Nonetheless, resistant strains may become an issue in the future [14]. Problems with the treatment of *etb*-positive community-associated MRSA (CA-MRSA) causing SSSS in healthy adults have already been reported in Japan [14,15].

Apart from antibiotic treatment, maintaining the body temperature and protecting the denuded skin to prevent secondary infections and fluid loss are also recommended [5]. SSSS predominantly affects neonates and infants, but immune system and renal impairment are reported to be susceptibility factors in adults. Mortality among treated children is low and does not exceed 5% [3,16]. The number of fatal cases in adults is much higher, reaching 59% in some studies [3]. The higher mortality in adults is explained by the fact that SSSS predominantly occurs with severe underlying disease. Single cases of SSSS have also been reported in adults with no obvious underlying disease [17,18].

SSSS is characterized by rare local outbreaks among neonates and sporadic occurrences in adults. For example, the French National Center of Staphylococcal Toxins estimated the number of cases at about 36 annually in the 1990s, while single outbreaks generally involve around a dozen of cases [19]. There are no data concerning the prevalence of SSSS over larger geographical areas.

## 3. Toxin Identity

The features of SSSS were first described by Baron Gottfried Ritter von Rittershain in 1878 [20]. However, it was not until 1967 that the relationship between skin exfoliation and *S. aureus* was determined by Lyell [21]. This significant delay was caused by the fact that the blister fluid and exfoliated regions are often free of cultivable staphylococci, because the toxin is distributed from distant sites of infection through the bloodstream. The existence of a hypothetical toxin was suggested by Lyell and confirmed by Melish *et al.* in 1972, who demonstrated the induction of blistering with sterile filtrates of bacterial cultures [22].

Early animal studies showed that blistering can be induced in mice with *S. aureus* strains isolated from patients with SSSS. It was demonstrated soon thereafter that the presence of bacteria is not necessary because blistering can be induced in model animals by a soluble factor found in the sterile filtrates of bacterial cultures. These early studies confirmed that a soluble toxin is solely responsible for all the pronounced disease manifestations. A reliable animal model was established, in which newborn mice inoculated with toxin producing strains or administered with sterile culture filtrates, reproduced the symptoms of human SSSS [23,24,25]. The toxin was subsequently purified and shown to be a protein of approximately 30 kDa [25,26,27,28,29]. It was soon shown that at least two serotypes of ETs exist, and these were designated ETA and ETB [30,31]. In Europe, USA, and Africa, ETA is prevalent, and is expressed by more than 80% of toxin-producing strains [3,32,33]. Only in Japan, are ETB-producing strains more prevalent than those expressing ETA [34,35]. Determination of the partial amino acid sequences of the purified toxins has allowed the corresponding genes to be cloned [36,37,38,39,40] and the toxins to be expressed in heterologous hosts. Recombinant toxins produced in *Escherichia coli* retained their activity in a mouse model, providing final confirmation that ETs are the sole factors responsible for blister formation in SSSS [39].

The orchestrated expression of multiple virulence factors is the key to the success of staphylococcal pathogenesis. The accessory gene regulator (*agr*) constitutes one of the major regulatory mechanisms described to date [41]. It has been demonstrated that the expression of both *eta* and *etb*, among many other virulence-factor-encoding genes, is regulated by *agr* [38,42]. Strains producing ETA and ETB show phylogenetic relatedness, as demonstrated on a representative group of 200 strains using amplified fragment length polymorphism (AFLP) analysis. ET-producing strains mainly belong to *agr* group IV [43,44].

## 4. Molecular Mechanism of Toxin Activity

Since the pioneering work of Melish in the early 1970s, the molecular mechanism by which ETs induce exfoliation remained a mystery. Epidermal detachment at the stratum granulosum was established by electron microscopy [45], but the direct mechanism remained unknown. Once the protein nature of ETs was established and the amino acid sequences determined [38,39,46,47], the close resemblance between the toxins and the serine proteases became immediately evident. Importantly, the catalytic triad residues of the chymotrypsin family proteases are well conserved in ETs [48]. Concurrently, it was proposed that peptide bond hydrolysis is the mode of the toxin action [48,49], but it took a decade to irrefutably demonstrate the biologically relevant proteolysis.

Since the resemblance of ETs to the serine proteases became apparent, multiple studies have tried to demonstrate their anticipated proteolytic activity. However, this proved much harder than initially expected, mainly because the ETs have one of the most limited substrate specificities found among known proteases. For this reason, early studies provided no direct evidence, whereas multiple indirect lines of supporting evidence were collected. Esterolytic activity (a common side activity of serine proteases) for the synthetic substrate Boc-GluOPh was reported [50], which provided a useful assay for ETs. The esterase activity of ETB was abolished with diisopropylphosphorofluoridate, a broad-range serine protease inhibitor [50]. The loss of esterase activity correlated with the loss of toxin effect in a murine model. Accordingly, a mutant at the serine of the catalytic triad, constructed in a heterologous expression system, lacked both esterolytic activity and epidermolytic activity when administered subcutaneously into mice [50,51,52]. Finally, a single biochemical study reported the hydrolysis of isolated peptides (alpha and beta melanocyte-stimulating hormones) by the purified toxin [53], but its physiological relevance was not demonstrated and the study was not confirmed by other authors at that time.

**Figure 1:**
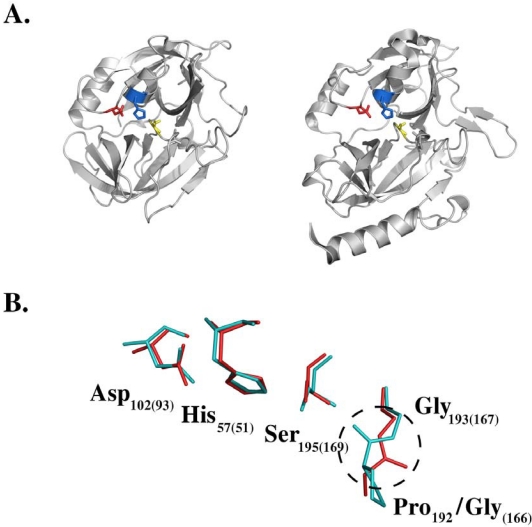
Exfoliative toxins belong to the chymotrypsin family of serine proteases and are structurally similar to staphylococcal glutamylendopeptidase (V8 protease). (**A**) Ribbon representation of the crystal structure of glutamylendopeptidase (left) and ETA (right). The catalytic triad residues Asp, His, and Ser are depicted in a stick model in red, blue, and yellow, respectively. Except for an additional helix characteristic of the exfoliative toxins and the conformation of some surface loops, the overall fold of both enzymes is almost identical. (**B**) The superimposition of the catalytic triad residues of glutamylendopeptidase and the corresponding residues of ETA, shown in red and light blue, respectively, demonstrates that this important part of the catalytic machinery is well developed in the toxin structure. Conversely, the oxyanion hole is not preformed in the structure of ETA, as demonstrated by the different orientations of the carbonyl oxygen of the Pro192-Gly193 peptide bond in ETA and the corresponding Gly166-Gly167 peptide bond in glutamylendopeptidase (dashed circle). The amino acid numbering is according to the Protein Data Bank (PDB) entries 1EXF (ETA) and 2O8L (glutamylendopeptidase; numbers in parentheses).

The overall picture was not at all consistent in the late 1990s, because multiple contradictory findings had also been demonstrated. For instance, broad-range serine protease inhibitors did not inhibit the exfoliation induced by ETA [48,54]. The crystal structures of both ETA and ETB were determined, and were almost identical to those of the serine proteases of the chymotrypsin family and specifically to that of the glutamic-acid-specific proteases. The conformation of the catalytic triad was preserved in both toxins [55,56,57,58], but the oxyanion hole was not preformed in either protein (Figure 1). The oxyanion hole constitutes an important part of the catalytic machinery of serine proteases, stabilizing the negative charge formed on a tetrahedral intermediate during catalysis. Therefore, the results of crystallographic experiments suggested that ETs are either proteolytically inactive or that an activation mechanism of some kind must exist. It was immediately speculated that the removal of the atypical N-terminal extension found exclusively in ETs, and not in other chymotrypsin-like proteases, was responsible. Although several studies suggested the proteolytic activation of the ETs, this was never convincingly demonstrated [48,50,51,59]. Moreover, the reports of different authors concerning the conformation of the oxyanion hole in ETB were conflicting [55,56,57,58]. Overall, until the very beginning of the 21st century, no direct evidence of the proteolytic activity of the ETs and especially its association with skin exfoliation was available, although multiple facts favored this hypothesis. 

Adding to the overall uncertainty concerning the mechanism of ET activity, another theory concerning the mode of toxin action was developed, concurrently with efforts to demonstrate its proteolytic activity. Based on information about other staphylococcal toxins, it was proposed that ETs function as superantigens, proteins that induce the atypical, polyclonal proliferation of T cells. In a classical way, the antigens processed by antigen-presenting cells are exposed as peptides bound to MHC-II molecules and selectively induce the proliferation of T cells, which specifically recognize the presented antigen via the T-cell receptor (TCR). Superantigens interact directly with invariant regions of MHC-II and TCR, inducing the antigen-independent proliferation of large populations of T cells, resulting in the deregulation of the immune response [60]. The initial results concerning the presumed superantigen activity of ETs were confusing and contradictory. Early reports by Morlock *et al.* [61] and Choi *et al.* [62] demonstrated the mitogenic activity of ETA purified from staphylococcal culture supernatants. Morlock *et al.* [61] assayed the activity in preparations of murine splenocytes, demonstrating that ETA interacts primarily with T cells and that its mitogenicity is similar to that of enterotoxin A. The study of Choi *et al.* [62] demonstrated the elevated expression of a particular variant of the gene encoding the TCR β chain in human and murine T cells after their interaction with ETA. Soon after, other researchers suggested that the results obtained by the groups of Morlock and Choi were the effects of sample contamination with trace amounts of enterotoxins, demonstrating that recombinant ETA isolated from superantigen-free strains of *S. aureus* or strains of *E. coli* had no mitogenic activity when assessed in human peripheral blood mononuclear cells and murine splenocytes. The same authors also demonstrated that the superantigenicity of commercial preparations of ETs could be attenuated with antibodies directed against enterotoxins A and B [63,64]. Nonetheless, later reports have indicated that ETs are truly superantigens and that their mitogenic activity is independent of their proteolytic activity. Vath *et al.* demonstrated that both the wild-type and a proteolytically inactive mutant toxin purified from superantigen-free *S. aureus* strains induced thymidine incorporation in human T lymphocytes [55]. Rago *et al.* produced mutants with modulated mitogenic activity. As a most striking example, the D146G mutation in the D-loop of ETA totally abolished its mitogenic activity [65]. The superantigen activity of highly purified ETA and ETB was also confirmed by Monday *et al.*, who showed the stimulated expression of specific TCR β chain genes in human T cells and mouse splenocytes after toxin treatment. The same authors pointed out that ETB had significantly higher pyrogenic activity than ETA in a rabbit model, where exfoliation was not induced and therefore the other effects of ETs could be easily distinguished. Nonetheless, both toxins showed milder effects than that of the classic superantigen TSST-1 [62,66]. Other researchers also confirmed the significantly lower mitogenic effect of the ETs compared with those of other superantigens [67]. Overall, it seems that if the ETs are truly superantigens (which remains controversial based on considerable contradictory evidence), their mitogenic properties are clearly weaker than those of other staphylococcal superantigenic toxins. Because SSSS lesions show no evidence of T-cell recruitment [5], the presumed superantigenicity of the ETs is probably not involved in the pathogenesis of SSSS.

## 5. Target of Exfoliative Toxins in the Skin

By the mid 1990s, it was strongly anticipated that ETs would prove to be proteases whose activity is manifested only under specific, as yet undermined, conditions. Their proteolytic activity seemed directly responsible for skin exfoliation while mitogenic activity, be it physiologically relevant or only observed under particular experimental conditions, was probably not directly associated with the primary manifestations of SSSS. The only significant missing piece of the puzzle at the time was the target molecule, the hydrolysis of which would induce skin exfoliation.

**Figure 2:**
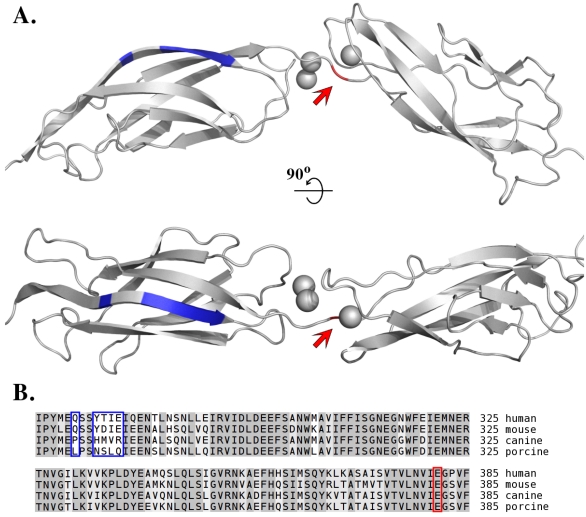
Exclusive specificity of exfoliative toxin A for human desmoglein 1 is dictated by primary interactions at the P1 specificity pocket and by secondary interactions with tertiary structural elements located away from the site of cleavage. (**A**) Homology model of domains EC3 and EC4 of human desmoglein 1 based on the crystal structure of domains EC3 and EC4 of *Xenopus laevis* C-cadherin (PDB ID: 1L3W). The glutamic acid residue determining the primary interaction at the P1 site of the enzyme and adjacent to the cleavage site is shown by the arrow (red). Distant sites of secondary interactions are marked in blue (according to [68]). Calcium ions, which stabilize the desmoglein structure and are essential for cleavage, are shown as grey spheres. (**B**) Sequence comparison of the EC3 domain of desmoglein 1 from different species explains the exclusive specificity of ETA for human and mouse desmoglein 1. Conserved amino acid sequences in the EC3 domains of the analysed species differ primarily in the region recognized by ETA. Colour coding as in panel A. (UniProt accession numbers for the desmoglein sequences: Q02413 human, Q7TSF1 mouse, Q9GKQ8 dog, Q3BDI7 pig).

The search for the specific target hydrolyzed by ETs was facilitated by studies of autoimmune diseases. Pemphigus foliaceus is characterized by disrupted cellular adhesions, leading to skin blistering and exfoliation, but does not affect the mucous membranes. The molecular basis of this phenomenon (acantholysis) is well established and involves auto-antibodies directed against desmoglein 1 (Dsg-1) [69,70,71]. Desmoglein 1 is a desmosomal cadherin [7,72,73] responsible for the integrity of those cell-to-cell adhesive structures. Because the clinical manifestations of pemphigus foliaceus are very similar to those of SSSS, it was hypothesized that Dsg-1 is the primary target of ETs. Accordingly, the hydrolysis of Dsg-1 (but not of other desmogleins) by ETA, ETB, and ETD was demonstrated experimentally both *in vitro* and *in vivo*, providing a final explanation of the mechanism of ET-induced epidermolysis [74,75,76,77]. These initial findings were followed by the detailed characterization of the mechanisms of Dsg-1 recognition and cleavage [68,77]. The cleavage sites were identified using the recombinant extracellular domain of Dsg-1 [74,75]. The previous assumptions, based mainly on crystallographic studies, concerning the substrate specificity of ETs for glutamic acid at the P1 sub-site (nomenclature according to Schechter and Berger [78]; “P1” signifies a residue adjacent to the scissile peptide bond towards the N-terminus of the substrate) [50,53,56,57,58], were directly confirmed [77]. It was also demonstrated that unlike classical serine proteases, this cleavage is highly dependent on the conformation of Dsg-1, and the unfolded protein is not hydrolyzed [79]. The folding of the extracellular domains of Dsg-1 depends on calcium ions [68,72,79]. The removal of calcium results in domain denaturation and the loss of the capacity of ETs to recognize and hydrolyze Dsg-1 [79]. The mechanisms of this precise recognition and specific cleavage were studied in molecular detail. Analysis of the ET interaction with domain-swapped variants of human desmoglein 1 (hDsg-1) and its canine counterpart (not hydrolyzed by ETs) identified the hDsg-1 region responsible for its recognition and precise protease positioning (extracellular domain EC2). Further detailed analysis of point mutants allowed the definition of particular desmoglein residues crucial for the interaction (Q271, 274YTIE277) [68] (Figure 2). Furthermore, it was demonstrated that the K213A mutant of ETA is inactive in a murine model, confirming the previous assumption that the residue determines the specificity of ETs for glutamic acid [65].

**Figure 3:**
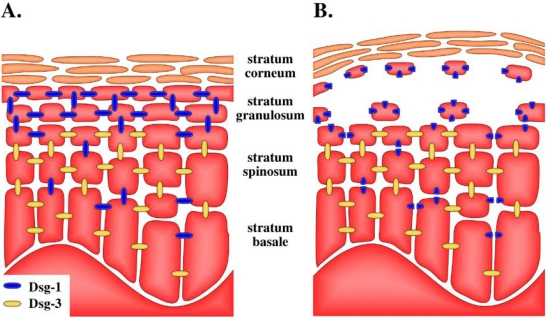
Differential distribution of desmoglein isoforms in the epidermis [80] explains the exfoliative-toxin-induced splitting at the stratum granulosum. Schematic representation of the desmoglein distribution in (**A**) healthy skin and (**B**) skin exposed to exfoliative toxin. In all strata, except the stratum granulosum, the exfoliative-toxin-mediated hydrolysis of desmoglein 1 (Dsg-1) is compensated by desmoglein 3 (Dsg-3). Dsg-3 is absent in the stratum granulosum, which explains the cell detachment and the splitting of the epidermal layers upon the hydrolysis of Dsg-1.

In SSSS, blistering affects only the superficial skin and not the mucosa or deeper skin layers. This phenomenon is elegantly explained by the selectivity of desmoglein cleavage and the differential expression of particular desmogleins in different layers of the skin and mucosa. The ETs selectively hydrolyze Dsg-1, whereas Dsg-3 remains unaffected. Dsg-1 is expressed in all strata of the skin, whereas Dsg-3 is only expressed in deeper strata [72,80]. Therefore, in the deep layers of the skin, the disruption of Dsg-1 by ETs is compensated by Dsg-3 and exfoliation only occurs in the stratum granulosum, where Dsg-3 is not present (Figure 3). The mucous membranes are characterized by different expression patterns of desmogleins. Dsg-1 is present in the superficial layers only, whereas Dsg-3 is found in all strata [7]. This explains the lack exfoliation of the mucous membranes. The cleavage of Dsg-1 is compensated equally by Dsg-3 in all layers. These conclusions have been further confirmed by studies of pemphigus vulgaris, an autoimmune disease characterized by the production of auto-antibodies directed against Dsg-3 and primarily affecting the mucous membranes [71].

## 6. Toxin Susceptibility

In humans, SSSS primarily affects neonates. The same effects are observed in mouse models, in which the animals are susceptible only until day seven of life [81]. The search for a likely explanation followed two paths, inferred from the known susceptibility factors in adults. It is well established that the impairment of the immune system, including pharmacological immunosuppression in autoimmune diseases, lymphoma chemotherapy [82], and AIDS [4,16,83,84], are risk factors for both SSSS and bullous impetigo in adult human subjects. It was hypothesized that cross-reactive antibodies are responsible for toxin neutralization [85]. Studies in adult mice confirmed that treatment with immunosuppressants increased their susceptibility to ETs-producing *S. aureus* strains. At the same time, no increased susceptibility to purified toxin was observed [17]. Studies in thymectomized mice demonstrated that the humoral response was not involved in toxin resistance. In this animal model, no difference in the time course of the development of toxin resistance or the level of resistance in adults was observed [81]. Therefore, it seems that the mechanism of resistance may differ in its details between humans and mice, as far as the involvement of the immune system is concerned.

Severe kidney disease is another susceptibility factor for SSSS in adults. Data are available that demonstrate that the toxin susceptibility of mice is dictated solely by the rate of its clearance from the bloodstream, and that the overall condition of the immune system has no effect on toxin susceptibility. Toxin clearance increases dramatically in the first week after birth, correlating with the development of resistance [81]. However, as mentioned above, it seems that in human subjects, unlike in mice, the overall condition of the immune system is also important. Renal impairment results in the deregulation of the immune responses [86,87,88], which may further increase the susceptibility to either the toxin itself or simply pathogen infection. It remains to be determined whether the impairment of renal clearance or the deregulation of the immune response is primarily responsible for toxin susceptibility in human subjects with underlying kidney disease.

## 7. Species-Specific Diversity of ETs

Since the discovery of exfoliative toxins ETA and ETB [5,31], multiple homologous toxins have been isolated from *S. aureus* and other species of staphylococci. It has been demonstrated that, together with the host specificity of particular strains or species of the pathogen, the toxins produced are also specific for various host organisms. Human-infecting strains of *S. aureus* produce mainly ETA and ETB (1.5% and 0.5% of isolates, respectively [89]), the genes of which are chromosome and plasmid located, respectively [36,90]. ETD toxin, encoded by a gene located within a 9.0-kb pathogenicity island (chromosomal site encoding virulence-associated factors), has also been described [76], but is less common than the other two toxins [89]. All these toxins induce exfoliation in human but also in a mouse model [25,76,91,92,93]. Nonetheless, it seems that at least some toxins are involved not only in SSSS and bullous impetigo but also in other cutaneous infections. ETD-producing strains are mainly isolated from furuncles or cutaneous abscesses, and not from SSSS [76,94]. However, the relevant data are too few to allow final conclusions to be drawn. The production of ETC was demonstrated in an *S. aureus* strain isolated from a horse with phlegmon. This toxin can affect horses, chicks, and suckling mice [93]. *Staphylococcus hyicus*, a species commonly isolated from pigs, produces multiple ETs, including SHETA, SHETB [95,96], ExhA, ExhB, ExhC, and ExhD [97,98]. The SHETA-encoding gene is chromosomally located, whereas the SHETB-encoding gene is located on a plasmid [99]. Both toxins trigger exfoliation in piglets and chicks but not in mice [93,100]. All four Exh toxins cause exfoliation in pigs, but only ExhA and ExhC also cause it in neonatal mice [97,98]. *Staphylococcus chromogenes* produces the SCET exfoliative toxin [101], which is implicated in the pathogenesis of exudative epidermitis in adult pigs, but also induces exfoliation in chicks [102]. Some pig isolates of *S. chromogenes* have also been shown to produce ExhB [103]. Canine strains of *S. pseudintermedius* produce a serotype of ET designated EXI. This toxin induces exfoliation in a mouse model [104]. Overall, it is anticipated that with more detailed studies, novel serotypes of ETs will be discovered in different species of staphylococci. These will be characterized by slightly divergent, but partially overlapping, ranges of affected species, a phenomenon associated with their adaptation to species-specific differences in the structures of desmogleins. Such adaptations are associated with yet another significant feature shared by ETs and many other staphylococcal virulence factors, their locations on mobile genetic elements. This feature allows the horizontal transfer and shuffling of genes between strains, accelerating strain adaptation and allowing host jumping. It has been demonstrated that the gene encoding ETA is located on an integrated 43.5-kb phage (designated ΦETA) and can transfer horizontally [105]. The *etb* gene is plasmid encoded [46] and is therefore also likely to transfer horizontally. Apart from the *etb *gene, the 38.2-kb pETB plasmid carries genes encoding other virulence factors [46].

## 8. Concluding Remarks

Most pieces of the exfoliative toxins puzzle are currently in place. The toxins are serine proteases with very limited substrate specificity. The target protein, desmoglein 1, is recognized both through an interaction at the classical P1 site and via additional features in the tertiary structure, located away from the site of hydrolysis. The cleavage of Dsg-1 results in the destruction of desmosomal cell-cell attachments in a superficial layer of the skin. Macroscopically, this manifests as epidermal detachment, the primary symptom of SSSS. The toxin can spread with the bloodstream and therefore not all lesions are infected. Overall, the destruction of the epidermal barrier facilitates the efficient progression of the infection.

Apart from this clear and seemingly complete picture, several issues await further clarification. First, in the light of multiple conflicting reports, the true nature of the superantigenic properties of ETs and their relationship to their pathogenesis remain to be determined. A careful, quantitative analysis that compares the effects of ETs and those of classical staphylococcal superantigens (TSST-1, enterotoxins) in both an animal model and isolated lymphocytes, substantiated with basic biochemical studies of TCR and MHC binding by the ETs, would convincingly address these issues, and such a study is eagerly awaited. Second, SSSS mainly affects newborn children rather than adults, and the reasons for this are still obscure. It is well established that in humans, the overall proficiency of the immune system is responsible because immunosuppression is a major risk factor for SSSS in adults. It has been hypothesized that cross-reactive antibodies developed in childhood [106] neutralize ETs before they reach the superficial skin layers. Nonetheless, clearly contradictory findings have been published, demonstrating that thymectomized adult mice are resistant to ETs [81]. Overall, it seems that the mechanism of resistance differs in its details between humans and mice, and this issue requires further clarification. Another interesting, as yet unanswered, question concerning ETs regards their presumed roles in staphylococcal skin infections other than SSSS and bullous impetigo. Many such infections are characterized by extensive tissue damage, which, aside of other known factors, may well be caused by the localized action of ETs. This presumed effect has not yet been studied beyond the fact that ETD-producing strains are often isolated from lesions other than SSSS [94]. Apart from the issues discussed above, which are directly relevant to the role of ETs in staphylococcal physiology, the mechanisms underlying the substrate recognition by these proteases are most interesting. Currently available data suggest that ETs recognize their substrates by both the classic P1 site interaction and significant secondary interactions involving the tertiary structural features of the desmoglein ligand. Because such secondary interactions are uncommon among serine proteases, it would be very exciting to define the molecular interaction between ETs and Dsg-1 in atomic detail. If the importance of these secondary interactions in the substrate recognition and the high substrate specificity of the exfoliative toxins is confirmed, ETs might prove ideal tools for processing appropriately constructed recombinant fusion proteins. A more detailed investigation of the interaction between ETs and Dsg-1 may facilitate the development of such a system.

Overall, we believe that although the main issues concerning ET activity are already well established, the system is worth further attention because interesting and meaningful results should be achieved with such studies.
